# Liposomal daunorubicin and dexamethasone as a treatment for multiple myeloma – the DD Protocol

**DOI:** 10.1590/S1516-31802005000600003

**Published:** 2005-11-01

**Authors:** Frederico Luiz Dulley, Rosaura Saboya, Vânia Tietsche de Moraes Hungria, Nadjanara Dorna Bueno, Fernando Gomes de Mello, Maria Tereza Frota, Carlos Sergio Chiattone, José Carlos Barros, Nair Sumie Mori, Daniel Sturaro, Maria Cristina Martins de Almeida Macedo, Roberto Luiz da Silva, Leila Maria Magalhães Pessoa de Melo, Cármino Antonio Souza

**Keywords:** Multiple myeloma, Daunorubicin, Dexamethasone, Drug therapy, Drug toxicity, Mieloma múltiplo, Daunorrubicina, Dexametasona, Quimioterapia, Toxicidade, Quimioterapia, Toxicidade de drogas

## Abstract

**CONTEXT AND OBJECTIVE:**

Liposomal daunorubicin has been used to treat hematological malignancies, including multiple myeloma (MM). The goal was to evaluate efficacy, side-effects and toxicity of liposomal daunorubicin and dexamethasone (“DD Protocol”).

**DESIGN AND SETTING:**

Prospective study at Sírio-Libanês, São Camilo, Brasil and Alemão Oswaldo Cruz hospitals.

**METHODS:**

Twenty consecutive patients with active MM received four cycles of liposomal daunorubicin intravenously for two hours (25-30 mg/m^2^/day) on three consecutive days per month, with oral dexamethasone (10 mg every six hours) on four consecutive days three times a month.

**RESULTS:**

The male/female ratio was 1:1 and median age 60. Nine patients were stage IIA, ten IIIA and one IIIB. The median from diagnosis to starting DD was 13 months. All patients received four cycles, except one. Fifteen had already received chemotherapy before DD. Responses of > 50% reduction in serum monoclonal para-protein were observed in six patients after first cycle (30%), six after second (30%) and four after third (20%), while four (20%) did not obtain this. Initially, 17 patients (85%) had anemia: 12 (70%) achieved correction. Progressive disease was observed in three patients (15%), while one had minimal response, four (20%) partial and 12 (60%) complete. Hematological toxicity was acceptable: three patients (15%) had neutrophils < 1,000/mm^3^; none had thrombocytopenia. Gastrointestinal toxicity was mild: nausea (10%), anorexia (15%) and no vomiting.

**CONCLUSIONS:**

This treatment has mild toxicity and good response rate. It may therefore be feasible before autologous bone marrow transplantation.

## RESUMO

**CONTEXTO E OBJETIVO:**

A daunorrubicina lipossomal tem sido usada no tratamento em várias doenças hematológicas malignas, incluindo mieloma múltiplo (MM). O objetivo deste estudo foi avaliar a eficácia, efeitos colaterais e toxicidade da daunorrubicina lipossomal and dexametasona no Protocolo DD.

**TIPO DE ESTUDO E LOCAL:**

Estudo prospectivo, realizado nos hospitais Sírio Libanês, São Camilo, Brasil e no Hospital Alemão Oswaldo Cruz.

**MÉTODOS:**

20 pacientes com MM ativo receberam daunoxome (25-30 mg/m²/dia) por três dias consecutivos, mensal, por quatro meses (total de quatro ciclos), e dexametasona, 10 mg a cada seis horas por quatro dias consecutivos (dia 1 - 4, 9 - 12 e 17 - 20), também mensal.

**RESULTADOS:**

A mediana entre o diagnóstico e o início do protocolo DD foi de 13 meses. Quinze pacientes receberam alguma quimioterapia anterior ao protocolo DD. Uma redução maior que 50% do pico monoclonal sérico foi observada em seis paciente após o primeiro ciclo do DD (30%), em seis pacientes após o segundo ciclo (30%), em quatro pacientes após o terceiro ciclo (20%) e em quatro pacientes não houve redução (20%). No início do protocolo, 17 pacientes (85%) apresentavam anemia e em 12 destes pacientes (70%) a anemia foi corrigida. Doença progressiva foi observada em três pacientes (15%), um apresentava resposta mínima, quatro pacientes (20%) apresentaram resposta parcial e 12 (60%) apresentaram resposta completa. A toxicidade hematológica foi aceitável. Toxicidade em trato gastrointestinal foi leve, consistindo em náusea (10%) e anorexia (15%), sem episódios de vômito.

**CONCLUSÃO:**

Este tratamento apresentou uma baixa toxicidade, uma boa taxa de resposta e pode ser usado previamente ao transplante de medula óssea autogênico.

## INTRODUCTION

Anthracyclines are commonly used for treating multiple myeloma (MM) and have been incorporated into a number of well-established regimens.^1–3^ The major mechanisms for resistance to daunorubicin in the treatment of MM include amplification or overexpression of the multidrug resistance 1 (MDR-1) gene, which codes for transmembrane P-glycoprotein (PGP). This latter is thought to pump several cytotoxic drugs out of cells, thus giving rise to the so-called classic MDR.^4,5^ MM is incurable by conventional chemotherapy regimens because of the rapid development of MDR.^6^

Liposomal encapsulation of anthracyclines is a potential method of drug targeting, thereby altering both the antitumor activity and side-effects profile. Liposomal daunorubicin (DaunoXome^®^) was developed in an attempt to increase the delivery of the drug to tumors and protect normal tissue from its toxicity.^7^ In addition, liposomal daunorubicin presents different pharmacokinetics, with a potential for reducing dose-limiting cardiotoxicity in comparison with conventional daunorubicin. Moreover, the pharmacokinetic profile of liposomal daunorubicin provides sustained plasma levels following short periods of infusion and thus offers a practical alternative to continuous infusion. Liposomal daunorubicin has been shown to cause mild toxicity to patients.

Dexamethasone has been included in several chemotherapy schedules for treating MM. It has significant efficacy that has been proven in reports in the literature.^1,8-10^ On the basis of this background, we decided to study the effectiveness of a combination of liposomal daunorubicin and dexamethasone (“DD protocol”) on our MM patients.

## OBJECTIVE

The goal of this phase II prospective study was to evaluate the efficacy, side effects and toxicity of liposomal daunorubicin and dexamethasone in 20 consecutive patients with MM.

## METHODS

Twenty consecutive patients with active MM were enrolled in the DD protocol. The male/female ratio was 1:1 and the median age was 60 years (range: 40-73 years). The Durie and Salmon staging system^11^ was utilized for all the patients. Nine of the 20 patients (45%) presented MM in stage IIA, ten (50%) was in stage IIIA and one (5%) was in stage IIIB. Fourteen of the 20 patients (70%) patients had the immunoglobulin G myeloma type (IgG), three (15%) had the immunoglobulin A myeloma type (IgA) and three (15%) had light-chain MM, of whom two (10%) were kappa and one (5%) lambda. The median length of time from diagnosis to starting the DD protocol was 13 months (range: 1-76 months). Fifteen patients (75%) had already received a median of 11 courses of some chemotherapy before DD (range: 5-43 courses). Of these, 10 patients (50%) had continued with progressive disease and five (25%) patients presented partial response to the previous chemotherapy. For five patients (25%), the DD protocol was their first-line therapy. Table 1 shows the patients’ characteristics.

**Table 1.** Characteristics of 20 patients with multiple myeloma enrolled in a phase II study on liposomal daunorubicin and dexamethasone (DD)

		Number of patients
Total		20
Male patients		10
Age		60 years (range: 40-73)
Myeloma type	Immunoglobulin G	14 (70%)
	Immunoglobulin A	3 (15%)
	Light-chain	3 (15%)
Staging of myeloma	Stage I	None
	Stage II	9 (45%)
	Stage III	11 (55%)
Prior therapy		15 (75%)
β_2_ microglobulin > 2.5 mg/l before DD protocol		11 (55%)
Creatinine > 2.0 mg/dl before DD protocol		None

The protocol proposed (the “DD protocol”) consisted of the administering of DaunoXome^®^ at a dose of 25 to 30 mg/m^2^/day, intravenously over a two-hour period, for three consecutive days every 30 days, for four months (four cycles). This was given in association with oral dexamethasone, 10 mg every six hours for four consecutive days three times a month (days 1 to 4, 9 to 12 and 17 to 20), every month. Table 2 shows the DD protocol.

**Table 2.** “ DD Protocol”: treatment scheme for multiple myeloma with liposomal daunorubicin and dexamethasone

Days	Drug/resting	Dose/time of administration	Route
1	liposomal daunorubicin dexamethasone	25-30 mg/m^2^/day for 2 hours 10 mg, every 6 hours	intravenous oral
2	liposomal daunorubicin dexamethasone	25-30 mg/m^2^/day for 2 hours 10 mg, every 6 hours	intravenous oral
3	liposomal daunorubicin dexamethasone	25-30 mg/m^2^/day for 2 hours 10 mg, every 6 hours	intravenous oral
4	liposomal daunorubicin dexamethasone	- 10 mg, every 6 hours	- oral
5 to 8	Resting		
9 to12	liposomal daunorubicin dexamethasone	- 10 mg, every 6 hours	- oral
13 to16	Resting		
17 to 20	liposomal daunorubicin dexamethasone	- 10 mg, every 6 hours	- oral
21 to 30	Resting		

An echocardiogram was performed before the first and after the last cycle. In order to monitor the response to the treatment, a complete evaluation of the disease was carried out before each cycle. The criteria utilized for defining the disease response are summarized in Table 3.^12^

**Table 3.** Criteria for defining the response to the “DD Protocol” in patients with multiple myeloma, in accordance with definitions from the European Bone Marrow Transplant group^12^

**1.**	**Complete response (CR)** a) Absence of the original monoclonal paraprotein b) Less than 5% of plasma cells in bone marrow, confirmed with bone marrow biopsy
**2.**	**Partial response (PR)** a) More than 50% reduction of the monoclonal paraprotein b) More than 50% reduction in plasma cells in bone marrow
**3.**	**Minimal response (MR)** a) Less than 50% reduction of the monoclonal paraprotein and plasma cells in the bone marrow
**4.**	**Progressive disease (PD)** a) No response to treatment

Tables 4 and 5 describe the chemotherapy that each patient had previously undergone, according to disease stage, paraprotein type and disease response to the DD protocol.

**Table 4.** Previous chemotherapy (paraprotein type and response to “DD Protocol”) of nine patients with stage II multiple myeloma

Number of patients	Previous chemotherapy courses	Paraprotein type and response to DD protocol
1	26 MP + 17 VBMCP	IgG – PR
1	10 VAD	IgG – CR
1	6 VAD	λ urine – CR
1	4 COMP + 1 VBMCP	κ – CR
2	6 COMP + 5 MP	IgG – CR, PD
3	None	κ – CR, IgG – CR, IgA – PR

*M = melphalan, P = prednisone, V or O = vincristine, B = bleomycin, C = cyclophosphamide, A = adriamycin, D = dexamethasone, IgG = myeloma immunoglobulin G type, IgA = myeloma immunoglobulin A type, λ = myeloma lambda type, κ = myeloma kappa type, PR = partial response, CR = complete response, PD = progressive disease.*

**Table 5.** Previous chemotherapy (paraprotein type and response to “DD Protocol”) of 11 patients with stage III multiple myeloma

Number of patients	Previous chemotherapy courses	Paraprotein type and response to DD Protocol
1	12 MP + 1 VBMCP	IgG – CR
1	12 MP + LPCV	IgA – PR
1	13 MP	IgG – PD
1	8 MP	IgA – CR
1	12 MP + 3 VAD	IgG – PD
1	MP	IgG – CR
3	MP + VBMCP + VAD	IgG – 2 CR, 1 MR
2	None	IgG – CR, PR

*M = melphalan, P = prednisone, V = vincristine, B = bleomycin, C = cyclophosphamide, A = adriamycin, D = dexamethasone, L = L-asparaginase, IgG = myeloma immunoglobulin G type, IgA = myeloma immunoglobulin A type, λ = myeloma lambda type, κ = myeloma kappa type, PR = partial response, CR = complete response, PD = progressive disease, MR = minimal response).*

### Statistical analysis

The statistical analysis was based on the data for the MM group in day/month/year format (D/M/Y). All data were analyzed using descriptive statistical methods, making use of the proportions of patients with each characteristic and outcome, including short-term side effects.

## RESULTS

**Figure 1:**
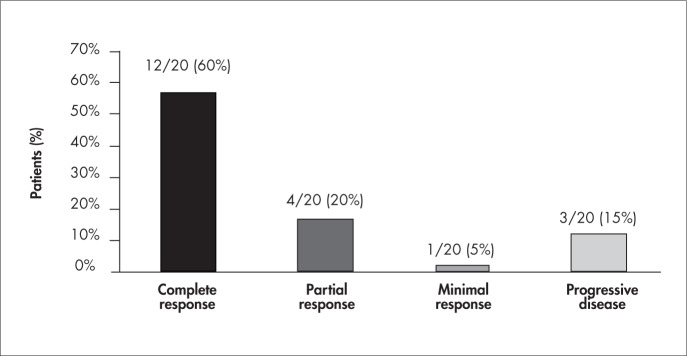
Responsiveness of 20 patients with multiple myeloma to a new protocol including dexamethasone and liposomal daunorubicin.

A reduction of more than 50% in serum monoclonal paraprotein was observed in six of the 20 patients (30%) after the first DD cycle, six patients (30%) after the second cycle and four patients (20%) after the third cycle, while four patients (20%) did not obtain such a reduction. Initially, 17 patients (85%) presented anemia, and 12 of these patients (70%) achieved correction by the end of the treatment protocol. All three light-chain MM patients are still alive and still presenting a complete response after time periods ranging from 10 to 31 months subsequent to undergoing the protocol. Of the three IgA patients, two had a partial response and one had a complete response, but all they relapsed and died of progression of the disease. Of the 14 IgG patients, eight (57%) had a complete response, two (14%) had a partial response, one (7%) had a minimal response and three (21%) had progressive disease. Five patients received the treatment as first-line therapy, of whom three achieved a complete response (two IgG and one kappa) and two a partial response (one IgG and one IgA). Overall, there was progressive disease in three patients (15%), a minimal response in one patient, a partial response in four patients (20%) and a complete response in 12 patients (60%), as shown in Figure 1. Eleven out of the twenty patients (55%) treated are still presenting a complete response after a median time period of 9 months (range: 3-31 months) subsequent to the treatment.

### Side-effects and toxicity

The hematological toxicity was very acceptable. Only three patients (15%) presented neutrophils < 1,000/mm^3^, and no patients had thrombocytopenia (defined as platelet count of less than 50,000/mm^3^). One patient presented a urinary tract infection and two others pneumonia. The gastrointestinal toxicity was mild, and it consisted of nausea (10%) and anorexia (15%), but without vomiting. Three patients (15%) had asthenia, and no cardiac abnormalities were observed and no lethal complication. No alopecia as a consequence of the DD protocol was observed.

## DISCUSSION

Anthracyclines are frequently utilized in the treatment of MM. Liposomal daunorubicin shows a potential for reducing dose-limiting cardiotoxicity, in comparison with conventional daunorubicin. Such cardiotoxicity is generally irreversible and refractory to medical therapy.^4,5^

This phase II study seems to confirm the efficacy of liposomal daunorubicin plus dexamethasone in patients with MM who have previously been treated or are receiving it as front-line therapy. Several of the most popular chemotherapy regimens utilized for treating MM^1,13^ involve continuous infusion of anthracyclines over several days. Liposomal daunorubicin provides sustained plasma levels following a short infusion and thus offers a practical alternative to continuous infusion.^14,15^

In the present study, by using liposomal daunorubicin we achieved an overall response rate of 80%, of which 60% was a complete response and 20% was a partial response. Most of our patients obtained a stable response after two cycles of treatment. All of the previously untreated patients presented some response: three out of these five patients achieved a complete and the other two obtained a partial response. These results are slightly better than in data published by other authors^8–10^ and suggest that the DD Protocol could be used as first-line therapy for this type of MM patients. However, few controlled studies have used liposomal daunorubicin in the treatment of MM.^8–10^ Mohrbacher et al.,^9^ using liposomal daunorubicin plus dexamethasone, demonstrated activity in bad prognosis MM patients that was comparable to the activity of standard regimens. In our study, the side-effects and toxicity related to liposomal daunorubicin were mild and easily controlled. Only three of the 20 patients (15%) presented neutropenia (counts of less than 1,000/mm^3^), and there were two cases of pneumonia that were treated with antibiotics. No cardiac abnormality was observed. No lethal complication has been observed so far.

## CONCLUSION

The DD protocol seems to be efficacious in MM patients, including those who have already undergone heavy treatment, and it can be used as first-line therapy. The protocol showed a good response rate and therefore might be feasible before autologous bone marrow transplantation.
